# Supplementary material for: Bicarbonate-Dependent Detoxification by Mitigating Ammonium-Induced Hypoxic Stress in Triticum aestivum Root
Source: Biology (Basel). 2024 Feb 5;13(2):101. doi: 10.3390/biology13020101 (PMC10886950; doi:10.3390/biology13020101)
Supplement: Supplementary file 1 [file biology-13-00101-s001.zip › biology-2838922-supplementary.pdf]

Table S1. Fresh weight of wheat seedlings grown in different strengths of Hoagland nutrient solution for 48 h.

Strength of Hoagland nutrient solution	Shoot (g $plant^{-1}$)	Root (g $plant^{-1}$)	Root/Shoot
1×	0.147 ± 0.008a	0.102 ± 0.011c	0.6939
1/2×	0.140 ± 0.007ab	0.108 ± 0.005c	0.7714
1/3×	0.131 ± 0.007b	0.098 ± 0.0021c	0.7481

Table S2. Quality assessment of RNA-Seq sequencing results.

Sample	Raw reads	Raw bases	Clean reads	Clean bases	Error rate	Q20 (%)	Q30 (%)	GC (%)
CK-1*	71263408	10.69G	68750162	10.31G	0.03	96.3	90.62	55.7
CK-2*	79141928	11.87G	76469550	11.47G	0.03	96.49	91.06	55.78
CK-3*	78414886	11.76G	75868586	11.38G	0.03	96.35	90.8	55.18
SA-1	76828216	11.52G	74101802	11.12G	0.03	96.72	91.55	54.71
SA-2	89181684	13.38G	85213634	12.78G	0.03	96.77	91.69	56.19
SA-3	76717974	11.51G	74203576	11.13G	0.03	96.35	90.75	54.95
AC-1	75958590	11.39G	73387174	11.01G	0.03	96.51	91.16	56.64
AC-2	88898670	13.33G	86450208	12.97G	0.03	96.53	91.15	55.23
AC-3	89463382	13.42G	85945562	12.89G	0.03	96.51	91.12	56.4

CK: 7.5 mM NO_3^- treatment; SA: 7.5 mM NH_4^+ treatment; AN: 7.5 mM NH_4^+ + 3 mM HCO_3^- treatment. *represents three biological replicates.
